# GNAI1 Suppresses Tumor Cell Migration and Invasion and is Post-Transcriptionally Regulated by Mir-320a/c/d in Hepatocellular Carcinoma

**DOI:** 10.7497/j.issn.2095-3941.2012.04.003

**Published:** 2012-12

**Authors:** Jian Yao, Lin-hui Liang, Yu Zhang, Jie Ding, Qi Tian, Jin-jun Li, Xiang-huo He

**Affiliations:** 1 State Key Laboratory of Oncogenes and Related Genes, Shanghai Cancer Institute, Renji Hospital, Shanghai Jiao Tong University School of Medicine, Shanghai 200032, China; 2 State Key Laboratory for Diagnosis and Treatment for Infectious Diseases, The First Affiliated Hospital, College of Medicine, Zhejiang University, Hangzhou 310003, China

**Keywords:** GNAI1, HCC, miR-320, migration, invasion

## Abstract

**Objective:**

To explore the role and regulation of guanine nucleotide-binding protein G(i), α-1 subunit (GNAI1) in hepatocellular carcinoma (HCC).

**Methods:**

Expression of GNAI1 in HCC samples was determined by qRT–PCR and immunohistochemical (IHC) staining. Huh-7 and SNU-387 cells stably expressing GNAI1 were established by the infection of lentivirus transducing unit containing GNAI1. siRNA against GNAI1 was transfected into SMMC-7721 cells to knock down the GNAI1 expression in HCC cells. Mir-320a/c/d mimics were transfected into SMMC-7721 and SK-Hep-1 cells and the expression of GNAI1 was determined by Western blot. The migration and invasion of Huh-7, SNU-387, SK-Hep-1 and SMMC-7721 cells were investigated by Transwell assays.

**Results:**

The GNAI1 protein was significantly downregulated in HCC samples without changes in its mRNA levels. GNAI1 could inhibit the migration and invasion of HCC cells *in vitro*. Further investigations indicated that GNAI1 was a target of miR-320a/c/d in HCC cells. Transwell assays demonstrated that these microRNAs could promote the migratory ability and invasivesess of HCC cells *in vitro*.

**Conclusions:**

GNAI1 is downregulated in HCC and inhibits the migration and invasion of HCC cells. This study is the first to investigate the role of GNAI1 in cancer. Regulation of GNAI1 by miR-320a/c/d indicates new therapeutic avenues for targeting HCC metastasis.

## Introduction

Heterotrimeric G proteins are crucial elements of multiple cellular signal transduction pathways that cause diverse biological outcomes^[^^1^^]^. G proteins comprise two signaling components: a Gα subunit bound to guanine nucleotides and a Gβγ dimer dissociated from Gα upon its activation. The Gα subunits can be broadly classified into four families, namely, Gαs, Gαi, Gαq/Gα11, and Gα12/13. The guanine nucleotide-binding protein G(i), α-1 subunit (GNAI1), also known as Gαi-1, belongs to the Gαi family, which also includes Gαi-2, Gαi-3, Gαo, and Gαz^[^^2^^]^. Gαi family members primarily function as inhibitors of adenylyl cyclase^[^^3^^]^. Furthermore, increasing evidence suggests that signaling through Gαi proteins can also regulate cell proliferation and differentiation^[^^4^^,^^5^^]^. In addition, GNAI1 is also involved in human platelet aggregation^[^^5^^]^, signaling by pancreastatin receptor in rat liver^[^^6^^]^, taxol resistance in human ovarian carcinoma cells^[^^7^^]^, and hydrogen peroxide-induced apoptosis in human lung cancer cells^[^^8^^]^. However, the biological functions of GNAI1 in hepatocellular carcinoma (HCC) remain unclear.

MicroRNAs (miRNAs) are a class of tiny, noncoding RNA molecules that have been found in a wide variety of eukaryotic organisms. Accumulating evidence suggests that miRNAs post-transcriptionally regulate gene expression by either directly degrading mRNA or indirectly repressing protein translation^[^^9^^]^. These molecules also play fundamental roles in various cellular and biological processes, including apoptosis^[^^10^^]^, cell differentiation^[^^11^^]^, cell proliferation^[^^12^^]^, development^[^^13^^]^, and metabolism^[^^14^^]^. Computational analyses have indicated that more than 30% of human genes may be conserved miRNA targets^[^^15^^]^, and a single RNA molecule may be targeted by multiple miRNAs^[^^16^^,^^17^^]^.

In the current study, the expression of GNAI1 in HCC and the effects of GNAI on HCC cells were determined. Moreover, the regulation of GNAI1 by microRNAs was also explored in HCC cells.

## Materials and Methods

### Cell culture

HEK293T, Hep3B, HepG2, Huh-7, PLC/PRF/5, SK-Hep-1, SMMC-7721, HCCLM3, and MHCC97L cells were cultured in Dulbecco’s modified Eagle’s medium (DMEM), and SNU-387 cells were cultured in Roswell Park Memorial Institute (RPMI)-1640 medium. All media were supplemented with fetal bovine serum to a final concentration of 10% and antibiotics, and cells were incubated at 37°C with 5% CO_2_.

### RNA extraction and quantitative real-time polymerase chain reaction (PCR)

Total RNA was extracted using TRIzol reagent (Invitrogen, CA, USA). Reverse-transcribed cDNA was synthesized with PrimeScript RT reagent kit (TaKaRa, Dalian, China). Real-time PCR was performed with SYBR Premix Ex Taq (TaKaRa).

### Vector constructs

The GNAI1 lentiviral expression vector pWPXL-GNAI1 was constructed by replacing the green fluorescent protein fragment of the pWPXL vector with GNAI1 open reading frame (ORF) sequence. The 3’ UTR sequence of GNAI1 was amplified from genomic DNA extracted from normal liver tissues and was cloned downstream of a cytomegalovirus promoter-driven firefly luciferase cassette in a pCDNA3.0 vector. Using the appropriate primers, PCR amplification of the GNAI1 3’ UTR sequence generated a series of p-Luc-UTR luciferase reporter vectors. The sequences of the wild-type (WT) and mutant (MT) 3’ UTRs were confirmed by sequencing.

### Lentivirus production and cell transduction

Viral packaging was performed in HEK293T cells after co-transfection of pWPXL-GNAI1 with the packaging plasmid psPAX2 and the envelope plasmid pMD2.G using Lipofectamine 2000 (Invitrogen). The viruses were harvested 48 hours after transfection, and the viral titers were determined. The target cells (1×10^5^), including Huh-7 and SNU-387 cells, were infected with 1×10^6^ recombinant lentiviral transducing units in the presence of 6 µg/mL polybrene (Sigma, MO, USA).

### Oligonucleotide transfection

The GNAI1–small interfering RNA (siRNA) duplexes and the miR-RiboTM miRNA inhibitors were designed and synthesized by RiboBio (Guangzhou, China). For each well of a six-well plate, the cells were transfected with 5 µL of miRNA inhibitor or siRNA (20 µM) using Lipofectamine 2000 (Invitrogen). Forty-eight hours after the transfection, the cells were harvested for transwell assays or luciferase reporter assays.

### *In vitro* migration and invasion assays

For the transwell migration assay, 5×10^4^ cells were placed in the top chamber of each insert (BD Biosciences, NJ, USA) without matrigel coating. For the invasion assay, 1×10^5^ cells were placed on the upper chamber of each insert, which was coated with 150 µg Matrigel (BD Biosciences, MA, USA). For both assays, the cells were trypsinized and resuspended in DMEM or RPMI-1640. Medium supplemented (700 µL to 900 µL) with 10% fetal bovine serum was injected into the lower chambers. The cells were incubated at 37°C as follows: 8 h for SNU-387 in the migration assays; 14 h for Huh-7 and SMMC-7721 in the migration assays; and 38 h for Huh-7, SNU-387, and SMMC-7721 in the invasion assays. Subsequently, any cells remaining in the top chambers or on the upper membrane of the inserts were carefully removed. After fixation and staining in a dye solution that contained 0.1% crystal violet and 20% methanol, the cells adhering to the lower membrane of the inserts were counted and imaged with an IX71 inverted microscope (Olympus Corp., Tokyo, Japan).

### Luciferase reporter assay

HEK293T cells were seeded in 96-well plates and transfected with a mixture of 50 ng p-Luc-UTR, 5 pmol negative control or miRNA mimics (GenePharma, Shanghai, China), and 5 ng Renilla following the recommended protocol for the Lipofectamine 2000 transfection system. After 48 hours of incubation, the firefly and Renilla luciferase activities in the cell lysates were measured using the Dual-Luciferase Reporter Assay System (Promega, Madison, USA).

### Western blot assay

The cell lysates were resolved with sodium dodecyl sulfate polyacrylamide gel electrophoresis, transferred to nitrocellulose membranes (Bio-Rad, Hercules, USA), and blocked in phosphate-buffered saline (PBS)/Tween-20 containing 5% nonfat milk. The membranes were incubated with antibodies against GNAI1 (Santa Cruz Biotechnology, CA, USA), glyceraldehyde 3-phosphate dehydrogenase (GAPDH) (KangChen Biotech, Shanghai, China), and β-actin (Sigma, St. Louis, USA). The antigen–antibody complexes were detected using enhanced chemiluminescence (Pierce, IL, USA).

### Immunohistochemical (IHC) staining

All tissue samples were fixed in phosphate-buffered neutral formalin, embedded in paraffin, and cut into 5-µm-thick sections. The tissue sections were deparaffinized, rehydrated, and microwave-heated in sodium citrate buffer (10 mM, pH 6.0) for antigen retrieval. The sections were then incubated with 0.3% hydrogen peroxide/PBS for 30 min and blocked with SuperBlock solution (Pierce, Rockford, USA). The slides were incubated with GNAI1 antibody (Santa Cruz) overnight at 4°C at the optimal dilution, labeled using EnVision HRP (mouse) kit at room temperature for 30 min, incubated with 3, 3’-diaminobenzidine liquid substrate (DAKO, Glostrup, Denmark), and counterstained with Mayer’s hematoxylin (DAKO). All sections were observed and photographed using Axioskop 2 microscope (Carl Zeiss, Oberkochen, Germany). The numbers of positively stained cells were scored independently by two senior pathologists using the following criteria: 0 indicates that less than 5% of the cells were stained positively; 1 indicates 5% to 24%; 2 indicates 25% to 49% ; 3 indicates 50% to 74% ; and 4 indicates more than 74%.

### Statistical analysis

The results are presented as mean ± standard error of the mean (SEM). The data were subjected to Student’s *t*-test (two-tailed and *P*<0.05 was considered significant) unless otherwise specified (χ^2^ test, linear regression).

## Results

### GNAI1 was frequently down-regulated in HCC at the protein level but not at the mRNA level

**Figure 1:**
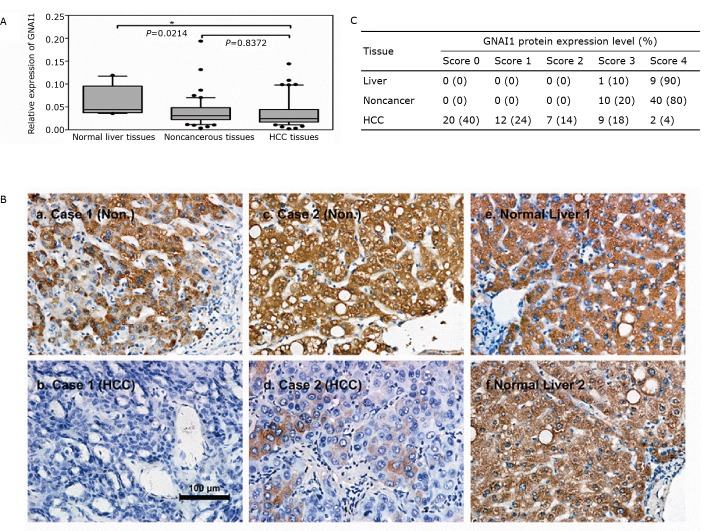
GNAI1 is frequently down-regulated in HCC at the protein level but not at the mRNA level. A: Relative expression levels of GNAI1 in HCC and normal liver tissues using qRT-PCR assays. The box-plot lines represent the medians and interquartile ranges of the normalized threshold values (Ct), and the whiskers and spots indicate the 1st to 90th percentiles and the rest of the data points; B: Relative expression levels of GNAI1 in 50 HCC cases and matched noncancerous liver tissues; C: IHC staining of GNAI1 in HCC and matched noncancerous liver tissues (Non.) or in normal liver tissues (Liver). The staining intensities were scored and are represented as follows: a) 3; b) 0; c) 4; d) 1; e) 4; and f) 4. Statistical analysis compared groups of 10 cases of normal liver tissues and 50 cases of HCC to groups of matched noncancerous liver tissues according to the scoring results.

To investigate whether the expression pattern of GNAI1 in HCC is similar to that of GNAI2, we measured both the mRNA and protein levels of GNAI1 in HCC samples and paired, adjacent noncancerous liver tissue samples from 50 patients as well as in normal liver tissue samples from 10 healthy patients. Although the mRNA level of GNAI1 was significantly lower in HCC than that in the normal liver samples (**Figure 1A**, *P*=0.0214), the GNAI1 mRNA levels showed no difference between the HCC and paired adjacent noncancerous liver tissues (**Figure 1A**, *P*=0.8372). However, an analysis of the GNAI1 protein expression in the HCC and normal liver tissues using IHC staining revealed that GNAI1 was significantly down-regulated in the HCC samples compared with the adjacent noncancerous liver tissues or the normal liver tissues (**Figure 1B**, HCC: *n*=50; normal liver: *n*=10). About two thirds of the HCC tissues (64%) had no or little expression of the GNAI1 protein. By contrast, the GNAI1 protein level was relatively high in all noncancerous and normal liver tissues (**Figure 1C**). The inconsistency between the GNAI1 expression at the mRNA and protein levels indicated the existence of post-transcriptional regulation, such as miRNA regulation. The GNAI1 expression in various HCC cell lines was also measured.

### GNAI1 significantly inhibited the migration and metastasis of HCC cells

**Figure 2:**
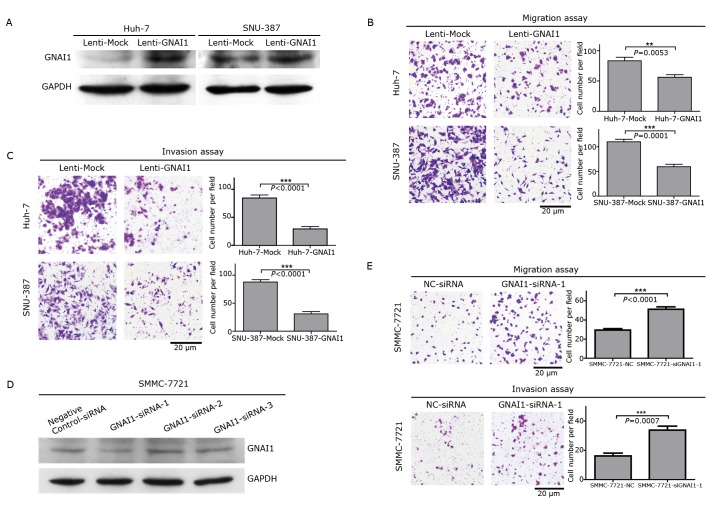
GNAI1 can significantly inhibit the migration and metastasis of HCC cells. A: Western blot of GNAI1 protein levels in Huh-7 and SNU-387 cells infected with lentiviral constructs containing GNAI1 or lentiviral constructs; B: Transwell migration assays of Huh-7 and SNU-387 cells stably overexpressing GNAI1 or the mock control. The representative images and quantification of the five randomly selected fields are shown. Values are shown as mean±SEM, along with the statistical significance; C: Transwell invasion assays of Huh-7 or SNU-387 cells expressing GNAI1 or the mock control; D: Western blot of GNAI1 expression levels in SMMC-7721 cells transfected with GNAI1-siRNAs or negative control-siRNA; E: Transwell migration and invasion assays of SMMC-7721 transfected with GNAI1-siRNA or negative control-siRNA.

We had previously reported that GNAI2 functions as an inhibitor of HCC migration and invasion. GNAI1 may possess biological functions similar to GNAI2 in HCC cells because both GNAI1 and GNAI2 are members of the Gαi family. To explore these functions, we established stable HCC cell lines by infecting Huh-7 and SNU-387 cells with a lentiviral construct that contained either the complete ORF of the GNAI1 gene or the vector alone (**Figure 2A**). The enhanced GNAI1 expression did not affect the growth of HCC cells. However, transwell assays without Matrigel indicated that GNAI1 could significantly suppress the migration of HCC cells compared with the vector groups (**Figure 2B**). Furthermore, transwell assays with Matrigel demonstrated that the invasiveness of the HCC cells was dramatically inhibited by GNAI1 compared with that by the vector groups (**Figure 2C**). To confirm whether GNAI1 can inhibit the migration and invasion of HCC cells, we knocked down the endogenous GNAI1 expression in SMMC-7721 cells with siRNA (**Figure 2D**). Consistent with the overexpression transwell assays, the knockdown of the endogenous GNAI1 significantly enhanced HCC cell migration and invasion (**Figure 2E**). Taken together, these observations indicate that GNAI1 is a negative regulator of HCC migration and invasion, similar to its family member GNAI2.

### A set of miRNA down-regulated the protein level of GNAI1 via 3’ UTR binding

**Figure 3:**
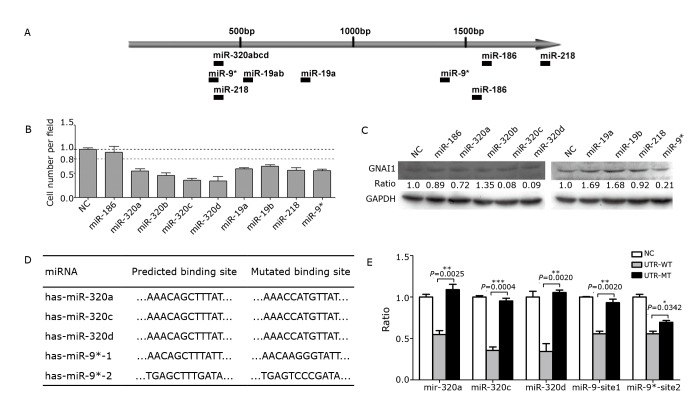
A set of miRNAs can downregulate the GNAI1 protein level by binding to its 3’ UTR. A: Schematic diagram of the predicted binding sites of the GNAI1-regulating miRNAs in the 3’ UTR of GNAI1. Several miRNAs shared the same target sites; B: Relative luciferase activity analyzed following the co-transfection of a luciferase reporter plasmid containing the WT GNAI1 3’ UTR and GNAI1-regulating miRNAs. Normalized to the Renilla luciferase activity, the data are shown as mean±SEM; C: Western blot assays of the endogenous GNAI1 protein levels in SMMC-7721 cells transfected with GNAI1-regulating miRNAs or negative control. The intensity was normalized to GAPDH, and the ratios indicate the relative expression levels compared with the negative control; D: Sequences of WT and MT (shadowed and underlined) binding sites; E: Relative luciferase activity in cells co-transfected with GNAI1-regulating miRNAs, and a luciferase reporter plasmid containing the WT or MT 3’UTR. The data are presented as mean±SEM, along with the *P* values.

Our previous experimental data has shown that the GNAI1 expression varies at the mRNA and protein levels, which may involve post-transcriptional regulation. Because microRNAs could down-regulate the protein levels of target genes without necessarily changing their mRNA expression, we examined whether miRNA was involved in the regulation of GNAI1. Using two internet-based miRNA target-prediction algorithms (TargetScan and PicTar), we identified nine potential binding sites (**Figure 3A**) for nine miRNAs in the 3’ UTR of GNAI1. We co-transfected the HEK293T cells with individual miRNA mimics and luciferase reporter plasmids to determine whether the predicted miRNAs were bound directly to the GNAI1 3’ UTR. The full-length wild-type GNAI1 3’ UTR was directly inserted into the region immediately downstream of a luciferase reporter gene. As shown in **Figure 3B**, all the predicted miRNAs, except miR-186, significantly reduced the luciferase activity. Furthermore, Western blot assays revealed that miR-320a/c/d and miR-9* dramatically down-regulated the endogenous GNAI1 protein level (**Figure 3C**), which indicates that these four miRNAs may directly target GNAI1 in HCC cells. To clarify whether these four miRNAs could bind directly to the 3’UTR of GNAI1, we analyzed the binding sites predicted by the algorithms and mutated the binding site sequences (**Figure 3D**). Reporter assays revealed that in the cells transfected with 3’ UTRs that contained mutations in the miR-320a/c/d or miR-9* binding site 1 but not site 2, the level of luciferase activity remained similar to that of the control cells (**Figure 3E**). This analysis indicated that these binding sites were authentic.

### MiRNA 320a/c/d remarkably facilitated HCC cell migration and invasion

**Figure 4:**
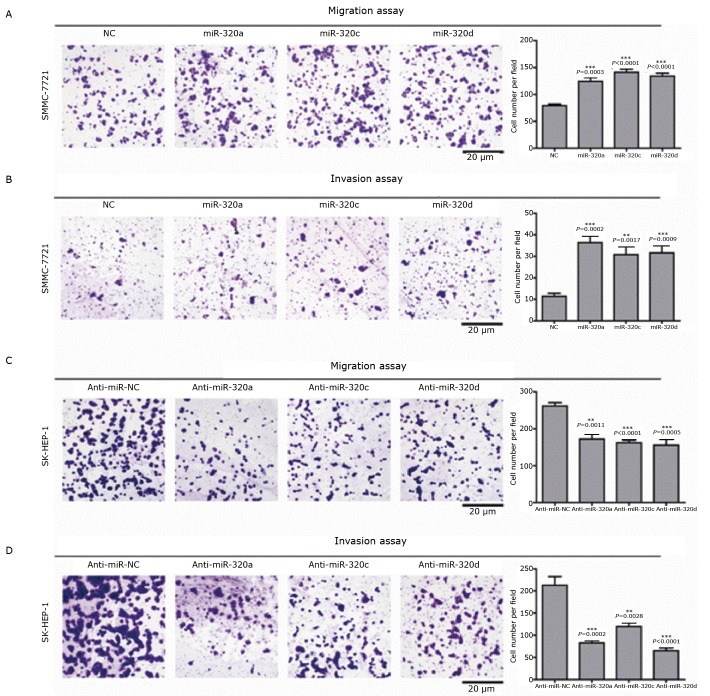
MiRNA-320 can remarkably facilitate HCC cell migration and invasion. A: Transwell migration assays of SMMC-7721 cells transfected with miR-320a/c/d or negative control. Representative images are shown, with the quantification of five randomly selected fields on the right. The values (presented as the means±SEM) and the statistical significance are shown; B: Transwell invasion assays of SMMC-7721 cells transfected with miR-320a/c/d or mock control. C: Transwell migration assays of SK-Hep-1 cells transfected with anti-miR-320a/c/d or anti-miR-NC; D: Transwell invasion assays of SK-Hep-1 cells transfected with anti-miR-320a/c/d or anti-miR-NC.

To further explore the biological functions of miRNAs in HCC cells, we ectopically expressed miR-320a/c/d or miR-9* in the HCC cell line SMMC-7721. The results showed that the overexpression of miR-320a/c/d significantly enhanced the migration and invasion of the HCC cells (**Figure 4A** and **4B**), whereas miR-9* overexpression produced no obvious effect (data not shown). By contrast, the downregulation of the endogenous miR-320a/c/d with specific inhibitors dramatically suppressed the migration and invasion of SK-Hep-1 cells (**Figure 4C** and **4D**). Taken together, these observations indicate that miR-320a/c/d robustly facilitate the migration and invasion of HCC cells.

## Discussion

In the current study, we found that the protein level of GNAI1 is frequently downregulated in HCC samples whereas its mRNA level remians unchanged. The *in vitro* transwell assays indicated that GNAI significantly suppressed the migration and invasion of HCC cells. Moreover, we found that GNAI1 is regulated by miR-320a/c/d, and miR-320a/c/d increased the migration and invasion of HCC cells *in vitro*.

GNAI1, a member of the Gα inhibitory family, transduces multiple extracellular signals to downstream molecules and participates in numerous physiological processes, such as proliferation^[^^18^^]^, adhesion^[^^19^^,^^20^^]^, and differentiation^[^^21^^]^. However, despite the physiological importance of GNAI1, its role in cancer remains unclear. In this study, we found that the GNAI1 protein level was downregulated in 64% of the HCC samples. To our knowledge, this study is the first to report on the downregulation of GNAI1 in cancer. Interestingly, the expression levels of GNAI2^[^^22^^]^ and GNAI3 (unpublished data) are also decreased in HCC. These results indicate that all the members of the Gαi family may be downregulated in HCC. Although the expression of the entire GNAI family has not been examined in other cancers, this phenomenon is worthy of investigation.

We explored the function of GNAI1 in HCC and found that GNAI1 did not affect the proliferation of HCC cells, although GNAI1 is reported to be involved in the cyclin D1 pathway^[^^23^^]^ and regulation of proliferation. We assume that this inconsistency is due to the regulation of different downstream pathways by GNAI1, depending on the cellular context. However, the *in vitro* transwell assays indicated that GNAI1 significantly inhibited the migration and invasion of HCC cells. Few studies have reported on the regulation of cell motility by GNAI1. However, only one study has reported that GNAI1 regulated the chemokine-induced migration of naive CD4+ T cells^[^^21^^]^. Our present findings uncover a new function of GNAI1 in cancer and extend the repertoire of the GNAI family. We previously found that GNAI2, another GNAI family member, also suppressed the migration and invasion of HCC cells^[^^22^^]^. In ovarian cancer cells, GNAI2 mediated lysophosphatidic acid-induced migration^[^^24^^]^. Collectively, these results suggest that the GNAI family plays an important role in cancer metastasis and may provide new therapeutic agents for cancer.

In exploring the mechanism of the downregulation of GNAI1 in HCC, we found that miR-320a/c/d could reduce the GNAI1 expression in HCC cells. The regulation of GNAI1 expression in cancer is rarely studied. Our finding that GNAI1 is suppressed by miRNAs in HCC indicates a new mechanism of GNAI1 regulation and suggests that the downregulation of GNAI1 in HCC cells might be caused by the upregulation of miR-320a/c/d. However, the expression of miR-320a/c/d in HCC remains unexplored, and future investigation of miR-320a/c/d expression will help to understand the downregulation of GNAI1 in HCC cells. Our results further showed that miR-320 promoted the migration and invasion of HCC *in vitro*, in contrast to the function of GNAI1, which further supports that the GNAI1 is the downstream functional target of miR-320a/c/d. The downregulation of GNAI1 by miR-320a/c/d might contribute to the metastasis of HCC. In addition, MiR-320 family is deregulated in numerous cancers^[^^25^^-^^27^^]^ and regulated the proliferation of cancer cells^[^^28^^]^, which hints that miR-320a/c/d might play an important role in the development of cancers. Together with the current results, miR-320 family could act as promising targets in cancer therapy.

In conclusion, GNAI1 may act as a suppressor for HCC metastasis and is regulated by miR-320a/c/d. It is worth to exploring the potential of the newly identified miR-320a/c/d-GNAI1 axis in the future anti-cancer therapy.
